# MEIBO (perfluorohexyloctane): a novel approach to treating dry eye disease

**DOI:** 10.1097/MS9.0000000000002322

**Published:** 2024-06-26

**Authors:** Ayesha Azhar, Muskan A. Taimuri, Malik Olatunde Oduoye, Anusha Sumbal, Ayesha Sheikh, Amna Iqbal, Areeba Ikram, Eisha Waqar

**Affiliations:** a Department of Internal Medicine, Dow University of Health Sciences, Karachi, Pakistan; b The Medical Research Circle, Gisenyi, Goma, Democratic Republic of Congo

**Keywords:** dry eye disease, Food and Drug Administration, keratoconjunctivitis sicca, Meibo (perfluorohexyloctane), perfluorohexyloctane

## Abstract

Dry eye disease (DED) or keratoconjunctivitis sicca (KCS) is a multifactorial disease that classically develops due to the hyperosmolarity of the tear film. Categorically divided into two types, based on decreased production and increased evaporation of the tear film, DED begins with a spectrum of nonspecific symptoms like pruritus, redness, burning and discomfort, progressively leading to stringy mucus eye discharge, photophobia, twitching, visual fluctuations, and punctate epithelial lesions. This disease has numerous treatment options, including medications, artificial tear inducers, and surgical manoeuvres that prevent water loss from the tear film. However, each of these treatment options has its limitations. The Food and Drug Administration (FDA) has approved another intervention, Meibo (perfluorohexyloctane), as a choice of management for dry eye disease. With its shielding action on the ocular surface, Meibo (perfluorohexyloctane) reduces desiccation stress-induced ocular damage, making it highly specific for treating DED. Available in an eye drop formulation of perfluorohexyloctane (PFHO), these drops can reduce saline evaporation by up to 80%. The methods we used for this analysis are literature searches from PubMed, Medline and Google Scholar. This study aims to scour varying differentials of DED, its aetiology, general interventions, the latest refinements, and clinical efficacy, safety, and trials associated with Meibo (perfluorohexyloctane) in the management of DED.

## Introduction

Highlights

- Dry eye disease (DED) or keratoconjunctivitis sicca (KCS) is a multifactorial disease that develops due to the hyperosmolarity of the tear film.
- Meibo reduces desiccation stress-induced ocular damage, making it highly specific for the treatment of DED.
- Available in an eye drop formulation of perfluorohexyloctane (PFHO), these drops can reduce saline evaporation by up to 80%.

Dry eye disease (DED) is a multifactorial ailment that possesses features common to that of an autoimmune aetiology, which classically presents as varying unspecific symptoms of eye discomfort. Dry eye disease, also known as keratoconjunctivitis sicca (KJS), causes damage to the ocular surface and is characterized by punctate epithelial lesions and symptoms such as burning, redness or stinging in the eyes. The pathophysiological event underlying DED is either a decrease in tear film formation or an increase in the evaporation of tears. Some available therapeutic interventions encompass artificial tear inducers, tear-stimulating medications, anti-inflammatory medications, and other surgical procedures^1^. Meibo (perfluorohexyloctane), the linchpin of our study, is yet another drug approved by the Food and Drug Administration (FDA), currently accessible in an eye drop formulation. Our study pertains to diving into multiple aspects of the ailment itself and its available treatment options, tapering it to the new advancements made in this regard, in the form of Meibo (perfluorohexyloctane), the most recent drug licensed for DED.

## Methodology

Sources of information and approach to searching: We looked through four databases—Pubmed, Google Scholar, Cochrane CENTRAL, and Clinical Trial. gov—for research demonstrating the effectiveness of Meibo (perfluorohexyloctane) for treating Dry Eye Disease (DED) from the beginning until 2023, without regard to language or time constraints. The search string is based on the medical subject headings (MESH) “Dry eye disease,” “Keratoconjunctivitis Sicca,” and “Meibo (perfluorohexyloctane).” To find relevant papers, a wide range of data sources were manually searched, including editorials, conference proceedings for indexed abstracts, bibliographies of the retrieved trials, meta-analyses, and systematic reviews.

## Epidemiology

Globally, the prevalence of dry eye disease (DED) varies from 5–34% with prevalence increasing with age. The large differences in prevalence figures of DED are due to variations in the definition, diagnostic criteria, classification, and study populations. Common risk factors for DED include age, female sex, postmenopausal oestrogen therapy, antihistamines, collagen vascular disease, corneal refractive surgery, irradiation, hematopoietic stem cell transplantation, vitamin A deficiency, hepatitis C, and androgen insufficiency^2^. Clinical data suggests that DED is more common in women with prevalence increasing from 5.7% among women less than 50 years of age to 9.8% among women aged over 75 years. Additionally, Hispanic, and Asian women are more likely to report severe symptoms compared with White women^3^.

## Pathophysiology and classification

The fundamental mechanism of DED is hyperosmolarity of tears, which is caused by low aqueous tear production and/or increased evaporation of tears and can subsequently result in inflammation and ocular damage^4^. The physiological range of tear film osmolarity is 296–302 mOsm/l; in the case of DED, the osmolarity rises to about 316–360 mOsm/l. Such an increase can activate the mitogen-activated protein kinases (MAPKs) and secretion of pro-inflammatory cytokines, chemokines, and matrix metalloproteinases in the tear film covering the ocular surface of the eyeball^1^. These mediators, along with hyperosmolarity induce apoptosis of corneal epithelium cells, conjunctiva, and goblet cells and damage the glycocalyx resulting in epithelial damage, diminished lubrication capacity of the ocular surface and instability of the tear film thus, exacerbating the hyperosmolarity of tears. This cycle of events is referred to as the ‘vicious circle’^5^.

Based on pathophysiology, the two main categories of DED include (a) aqueous-deficient dry eye where the rate of tear evaporation is normal but lacrimal secretion is reduced, and (b) evaporative dry eye where the rate of lacrimal secretion is normal but there is increased tear evaporation. The aqueous-deficient category can be further classified into Sjögren syndrome dry eye where inflammation of the lacrimal gland causes decreased secretion of the aqueous part of the tear film, and non-Sjögren syndrome dry eye, which can result from androgen hormone deficiency, lacrimal duct obstruction, or systemic drugs that lower body secretions. The evaporative dry eye also includes two further subcategories based on the underlying cause; intrinsic and extrinsic. Intrinsic causes include Meibo (perfluorohexyloctane)mian gland dysfunction, disorders of lid aperture, and low blink rate, while extrinsic causes include vitamin A deficiency, topical drugs preservatives, contact lens wear, and ocular disease^1^.

## Signs and symptoms of DED

In DED, the person exhibits a variety of symptoms that are typically bilateral. However, discomfort, burning, or scratching are the most common sensations that are also responsible for damaging the cornea by triggering an inflammatory response^1^. Other symptoms include stringy eye mucus, photophobia, excessive blinking and twitching, vision fluctuation, trouble wearing contacts, trouble seeing when driving at night, and eye fatigue^1,2^. Symptoms in DED may be brought on by one of two circumstances: either an increase in tear evaporation or decreased tear secretion. Watery eyes, however, are a frequent symptom seen in DED since it is the body’s response to make up for the Meibomian gland’s malfunction^2^.

## Current treatment options for DED

### Artificial tears

For most people, daily usage of over-the-counter eye drops, that function as artificial tears, is all that is required to treat the infrequent or mild symptoms of DED. Along with providing the natural tear components needed by the eye, artificial tears dilute the inflammatory cytokines, reduce tear hyperosmolarity and reduce the ocular surface’s sensitivity to inflammation^1^. Hydroxypropyl cellulose eye inserts, such as those found in the medication Lacrisert, also have a similar mechanism of action to artificial tears. Since most multidose artificial tears commonly include preservatives such as cetrimide, chlorobutanol, and benzalkonium chloride, they degrade gradually over time, dispensing a lubricatory effect to combat dryness in the eye^1^. However, when used for an extended period, this has the drawback of leading to toxic epitheliopathy^1^.

When no treatment is effective and DED is severe, eye drops manufactured from your blood serum may also be utilized as a replacement for natural tears. Blood serum consists of a variety of substances, including components of natural tears such as the proteins albumin and lactoferrin, immunoglobulins, vitamin A, enzymes, and growth factors, which can provide lubrication and promote healing of the ocular epithelium^1^. However, using autologous serum carries its setbacks such as the potential for contamination and the requirement for patient blood sample collection during each preparation period^1^.

### Anti-inflammatory medication

Anti-inflammatory medicine is used in moderate to severe cases of DED. These medications can alleviate the signs and symptoms of DED by reducing inflammation in the eyelids or the cornea^2^. Several different classes of anti-inflammatory drugs have been used for the treatment of DED, some of which are described below.

#### Cyclosporine

A peptide generated from a fungus called cyclosporine A (CSA) has anti-inflammatory and immunosuppressive properties. Topical cyclosporine A (0.05%) enhances goblet cell density, corneal fluorescein staining, and the Schirmer test value. However, the use of this medication is frequently accompanied by eye irritation when used for DED^1^.

#### Corticosteroids

Corticosteroids can be used to treat moderate to severe dry eye illness; however, long-term therapy will likely cause undesirable side effects to manifest such as cataract formation and increased intraocular pressure. Hence, this type of treatment is only advised for short-term therapy^1^.

#### Tetracyclines

Tetracycline and its derivatives such as doxycycline and minocycline have both bacteriostatic and anti-inflammatory properties, thus making them an effective treatment for the signs and symptoms of DED. They have been successfully used orally in small doses to treat corneal ulcers and dysfunction of the Meibomian gland. However, negative effects on the skin and digestive system might occur when used in high dosages^1,2^.

#### Fatty acid supplements

The polyunsaturated essential fatty acid omega-3, when used orally, can combat the effects of the primary inflammatory metabolite in the body; arachidonic acid; a long-chain omega-6 fatty acid. Other than their anti-inflammatory effects, omega-3 fatty acids like docosahexaenoic acid and eicosapentaenoic acid can improve the structural integrity of the retina, and reduce the signs and symptoms of DED^6^. A study from 2005 demonstrated a higher incidence of DED in women whose dietary intake of omega-3 fatty acids was lower than required^6^. Although increasing omega-3 fatty acid intake has the potential to reduce ocular inflammation in DED, oral supplementation may also raise the risk of prostate cancer^1^.

### Macrolides

Macrolides such as Azithromycin can be especially effective when DED is due to a bacterial Meibomian gland infection. As demonstrated in a previous study, the lipid layer secretion and stability of the tear film can be enhanced by using azithromycin eye drops to treat blepharitis and Meibomian gland dysfunction, which are of the primary causes of DED^1,2^.

### Tear-stimulating medication

Since one of the most common causes of DED is decreased secretion of tears, any medication that acts as a tear stimulant could be an effective method to mitigate the signs and symptoms of DED. Tear production may be boosted by cholinergic medications like pilocarpine and cevimeline; however, they might produce increased sweating as an adverse effect^7^. Tear production may also be enhanced by mucin secretagogues, such as diquafosol, which acts as a P2Y2 purinergic receptor agonist that stimulates conjunctival epithelial cells to secrete water, while also stimulating receptors in ocular tissues^1^. Additionally, a nasal spray called varenicline (Tyrvaya), which was recently approved by the Food and Drug Administration (FDA) to boost tear production, might also prove effective^8^.

### Other procedures

Some non-pharmacologic treatment methods have also been identified for the treatment of DED. For extremely severe aqueous-deficient DED, the tear ducts can be blocked using removable microscopic collagen or silicone plugs known as punctal plugs^2^. Blockage of the tear ducts prevents drainage of the tears through the puncta, which leaves the eye lubricated for a longer time and alleviates the symptoms of DED^2^. Another method of tear duct blockage is thermal cautery, whereby the application of heat to the puncta permanently blocks the duct and prevents tear drainage^9^. Although not cost-effective, some people who suffer from severely dry eyes may also choose to wear specialized scleral contact lenses, which are designed to shield the eye’s surface and trap moisture^1^.

## Mode of action

The US Food and Drug Administration (FDA) recently approved the novel topical medication therapy NOV03 (MEIBO (perflouohexyloctane)) for the management of the signs and symptoms of DED^10^. It is an eye drop made of perfluorohexyloctane (PFHO), an anhydrous semi-fluorinated alkane, without any water or preservatives^11^. Because PFHO is an amphiphilic molecule with different physical characteristics from protonated carbon chains and lower cohesive energy, it has lower surface tension than other alkanes^11^. The eye drop quickly spreads throughout the surface of the eye because of its minimal surface tension, forming a durable protective layer at the tear film-air interface that stops the aqueous phase of the tear film from evaporating and lessens the stress placed on the eyelid when blinking^12^.

Due to the similar refractive index of PFHO to water and its small drop size of about 10 ml, the eye is not overfilled after putting the drop, and residual time on the eye is prolonged without impairing vision^12^. Studies examining perfluorohexyloctane’s effects revealed that when it was applied over saline, the rate of evaporation of the solution was reduced by about 80%. This finding suggests that perfluorohexyloctane likely forms a layer on the tear film surface to stop evaporation, potentially enhancing the function of the tear film’s lipid layer by causing its reorganization and subsequent stabilization^13^. By preventing tear film evaporation, Meibo (perfluorohexyloctane) can reduce the risk of drying and hyperosmolarity, as well as apoptosis and inflammation of the ocular surface, all of which contribute to the signs and symptoms of DED^14^.

Meibo (perfluorohexyloctane) potentially reduces desiccation stress-related ocular surface inflammation and speeds up corneal healing. Therefore, applying perfluorohexyloctane on the eye causes tear turnover through blinking and is anticipated to restore and spread the tear aqueous layer long before tear evaporation dries the ocular surface^11^. The inflammatory response at the surface will be reduced by a restored tear film, which will then allow the corneal surface epithelium to heal. Additionally, it has also been reported that the lubricating action of MEIBO (perfluorohexyloctane) on the ocular surface may offer symptomatic relief to patients with dry eyes^11^. In Vittitow *et al*.^11^, it was observed that the treatment with perfluorohexyloctane resulted in a significant change from the starting point in the dryness score and had significant effects on the visual scale correlating to burning and other dry eye symptoms, including a foreign body sensation, irritation, sensitivity to light, and discomfort.

## Efficacy

### Randomized control trials

Five double-masked, one single-masked and one parallel-group monocentric, multicenter, randomized controlled trials were conducted. A total of 2289 patients were part of these trials in which perfluorohexyloctane ophthalmic drop, was administered 4 times daily. All the trials aimed to find out the effect of perfluorohexyloctane drops in treating dry eye disease (DED) and Meibomian gland dysfunction. After a follow-up of 4–8 weeks, the drug was found to successfully alleviate the symptoms of DED. In the first trial, an improvement from baseline in both total Corneal Fluorescein Staining (tCFS) score [(least square mean treatment difference (LSMD) 0.97 (–1.40, –0.55);*P*<0.001] and visual analogue scale dryness (VASD) score [LSMD –7.6 (–11.8, –3.4);*P*<0.001], both meeting primary efficacy endpoints^13^. In the second trial use of the drops showed improved tCFS [LSMD −1.2 (−1.7, −0.8); *P*<0.001] and VASD score [LSMD −10.2 (−14.4, −6.1);*P*<0.001]^15^. Similarly, the third randomized controlled trial (RCT) also yielded an increased tCFS score [mean (SD)−3.8 (2.7) vs.−2.7 (2.8)] and eye dryness score [mean (SD)−38.6 (21.9) vs. −28.3 (20.8)] in patients receiving perfluorohexyloctane^16^. The group receiving perfluorohexyloctane showed significant alleviation in symptoms including pain (*P*=0.003). In the fourth trial, tear film thickness and lipid layer thickness significantly increased over time with a maximum effect at the end of the study (LSMD: 6.42%; *P*=0.0142)^12^. One RCT showed an increase in lipid layer thickness from 45.8±8.8 ICU at baseline to 66.7±19.5 ICU at 12 weeks (*P*=0.002)^17^. Also, there was an increase in non-invasive tear break-up time from 12.4±5.9 16.9±4.7 s (*P*=0.02) after 12 weeks^17^. In the other trial, there was an improved tCFS −2.11 (−2.59 to −1.63) and Dryness Score −31.57 (−36.81 to −26.33) in the group receiving NOV03 4 times a day^18^. Another trial also found improved tCFS score [mean (SD), −3.8 (2.7) vs. −2.7 (2.8)] and eye dryness score [mean (SD), −38.6 (21.9) vs. −28.3 (20.8)], along with alleviation in the eye pain [mean (SD) tCFS score, 26.7 (23.7) vs. −18.7 (22.5); *P*=0.003] in the group receiving perfluorohexyloctane^19^. (Detailed information on RCTs in Table 1)

**Table 1:** Characteristics and results of clinical trial.

Name of study	Study design	Study population	Drug used	Follow-up for clinical efficacy	Efficacy	Safety	Adverse effect
Tauber (2023) *et al.* ^13^	Multicenter, double-masked randomized controlled trial (Phase 3)	597 participants (NOVO3, *n*=303; saline, *n*=294)	NOV03 eye drops (perfluorohexyloctane)	8 weeks	Improved tCFS score [least square (LS) mean treatment difference was –0.97 (95% CI: –1.40, –0.55; *P*<0.001)] VAS dryness score [–7.6 (95% CI: –11.8, –3.4; *P*<0.001).] meeting both primary efficacy endpoints.	Ocular AEs were experienced by 9.6% of patients.	Eye irritation, conjunctivitis, dry eye, punctate keratitis, blurred vision (mostly mild and transient), instillation site pain, and eye discharge.
Sheppard *et al.* ^15^	Double-masked, randomized controlled trial	620 participants (NOVO3, *n*=311; saline, *n*=309)	NOV03 eye drops (perfluorohexyloctane)	8 weeks	tCFS (−2.3 vs. −1.1; LS mean treatment difference, −1.2 [95% CI −1.7 to −0.8]; *P*<0.001) Visual analogue scale dryness score (−29.4 vs. −19.2; LSMD, −10.2 [95% CI −14.4 to −6.1]; *P*<0.001)	Ocular AEs were experienced by 12.9% of patients.	Blepharitis, conjunctival hyperaemia, conjunctival papillae, ocular hyperaemia, blurred vision, hordeolum (stye), and visual acuity reduction.
Tian *et al.* ^16^	Multicenter, double-masked randomized controlled trial (phase 3)	312 participants	Perfluorohexyloctane eye drops (SHR8058)	57 days	tCFS score (mean [SD], −3.8 [2.7] vs. −2.7 [2.8]) Eye dryness score (mean [SD], −38.6 [21.9] vs. −28.3 [20.8]) in the perfluorohexyloctane group Alleviated pain (mean [SD] tCFS score, 26.7 [23.7] vs. −18.7 [22.5]; *P*=0.003) Awareness of DED symptoms (mean [SD] tCFS score, −38.1 [25.1] vs. −23.7 [27.6]; *P*<0.001), Frequency of dryness (mean [SD] tCFS score, −43.3 [23.8] vs. −29.1 [24.8]; *P*<0.001).	Ocular AEs were experienced by 21.8% of patients	NA
Habbe *et al.* ^17^	Monocentric, parallel-group, randomized controlled trial	62 participants	Perfluorohexyloctane eye drops (F6H8)	12 weeks	Increased LLT from 45.8±8.8 ICU at baseline to 66.7±19.5 ICU at 12 weeks (*P*=0.002) Increased NIBUT from 12.4±5.9 seconds to 16.9±4.7 s (*P*=0.02) after 12 weeks.	NA	NA
Tauber (2021) *et al.* ^18^	Multicenter, double-masked randomized controlled trial (phase 2)	336 participants (NOVO3, *n*=225; saline, *n*=111)	NOV03 eye drops	Three follow-up visits at 2, 4 and 8 weeks, respectively	Decreased tCFS −2.11 (−2.59 to −1.63) in the NOV03 QID group, decreased tCFS −1.78 (−2.27 to −1.30) in the NOV03 BID group Dryness Score −31.57 (−36.81 to −26.33) in the NOV03 QID group, Dryness Score −30.43 (−35.75 to −25.11) in the NOV03 BID Group	Ocular AEs were experienced by 26.2% of patients	Blurred vision, eye pain, eye irritation
Tian *et al.* ^16^	Multicenter, double-masked randomized controlled trial (phase 3)	312 participants (perfluorohexyloctane group *n*=156; saline, *n*=156)	Perfluorohexyloctane eye drops	57 days	tCFS score (mean [SD], −3.8 [2.7] vs. −2.7 [2.8]) eye dryness score (mean [SD], −38.6 [21.9] vs. −28.3 [20.8] alleviated eye pain (mean [SD] tCFS score, 26.7 [23.7] vs. −18.7 [22.5]; *P*=0.003) awareness of DED symptoms (mean [SD] tCFS score, −38.1 [25.1] vs. −23.7 [27.6]; *P*<0.001) frequency of dryness (mean [SD] tCFS score, −43.3 [23.8] vs. −29.1 [24.8]; *P*<0.001)	Ocular AEs were experienced by 21.8% of patients	Blurred vision, ocular paraesthesia, eye pain, vision loss, foreign body sensation

AE, adverse effect; BID, twice a day; DED, dry eye disease; LLT, lipid layer thickness; LSMD, least square mean treatment Difference; NIBUT, non-invasive tear break-up time; QID, four times a day; tCFS, total corneal fluorescein staining; VAS, visual analogue scale.

### Pre-clinical study

A 7-day pre-clinical study was performed to determine the efficacy of perfluorohexyloctane for treating DED^20^. The adjusted volume spread area of perfluorohexyloctane was almost 2.5-fold greater than that of saline (*P*<0.001). The percentage increase in tear evaporation rate at the 5-minute time point was significant (*P*=0.044) for the perfluorohexyloctane group^12^.

### Observational study

Two prospective, multicenter, observational studies for patients of DED receiving perfluorohexyloctane in routine clinical care were performed. In the first study, 60 patients were enroled, and results showed improved tear film break-up time (8.72 ± 4.58 s), Ocular Surface Disease Index (OSDI) (26.37 ± 16.53; *P* <0.0001), and corneal+conjunctival staining sum (CSS) score (1.84 ± 1.58; *P*<0.0001)]^21^. At the end of the other study, tear secretion and tear film stability improved significantly (*P*<0.0026) in the 30 patients enroled in the study^22^.

## Safety and tolerability

In all the randomized clinical trials, the occurrence of the adverse ocular event was 9–22%. In the multi-centred observational study, only 9 out of 90 patients reported any adverse outcome. Whereas no adverse ocular event was seen in the pre-clinical study performed. The drug appears to be safe and well-tolerated within reported clinical studies. However, since these studies were conducted in a well-controlled environment these results may not depict the actual rate of adverse outcomes seen during practice.

The most common adverse events, as reported in a randomized controlled trial on 620 patients with a history of DED, were ocular and conjunctival blepharitis, blurred vision with reduced visual acuity, itching, and irritation in the eye^15^. Some patients experience conjunctivitis and punctate keratitis^21^. Also, a few patients reported complaints of red eyes, clotted eyes, and stingy mucus. Serious adverse reactions were seen in only five patients for whom the treatment was discontinued. These serious adverse reactions included foreign body sensation, application site reaction and hypersensitivity, sprain of ligament, ocular paraesthesia, and severe pneumonia^21^ (Details mentioned in Table 1).

Although perfluorohexyloctane seems to have a strong safety profile, there seems to be very little information available regarding its pharmacokinetics since it has not been extensively studied in humans. In vitro, it was not metabolized by the human liver microsomes, and a pharmacokinetic study showed low levels of perfluorohexyloctane in the blood following topical administration over the ocular surface^10^. More research is urgently required into the pharmacokinetics of the drug to make it a safer option for use.

## Future applications and limitations

The FDA’s approval of Meibo (perfluorohexyloctane) represents a significant step forward in the treatment of DED, one of the most prevalent ocular surface disorders, by addressing a huge unmet need for the millions of people who have the disease. A significant challenge in DED’s treatment is the evaporation of tears from the surface of the eyes, which is a major cause of DED^2^. However, with the approval of Meibo (perfluorohexyloctane), opticians can now adopt a new strategy for DED therapy with a first-of-its-kind, water-free, preservative-free prescription treatment option that focuses on tear evaporation specifically.

All the trials behind Meibo (perfluorohexyloctane)’s approval by the FDA reported significantly improved results, such as an increase in tear film thickness, tear film stability and a slower rate of ocular damage^12^. However, the previous studies regarding perfluorohexyloctane’s effect on DED had some limitations, such as the exclusion of patients with severe DED, a fairly brief treatment duration, the initial evaluation occurring no earlier than 2 weeks, and the lack of use of the identical dropper bottle for both the perfluorohexyloctane and hypotonic saline control to maintain study masking, which could have prevented a precise assessment of the duration of action of Meibo (perfluorohexyloctane)^11^.

## Conclusion

Our study reviewed the literature available on dry eye disease its available treatment modalities and how a new drug, Meibo (perfluorohexyloctane), can potentiate the prognosis of DED. Although a few side effects were reported in a small population, the efficacy and safety profile of perfluorohexyloctane is relatively strong. However, for a more detailed pharmacokinetic picture of the drug, long-term studies of Meibo (perfluorohexyloctane) treatment in patients with DED are required with relatively larger sample sizes.

## Ethical approval

Not applicable.

## Consent

Not applicable.

## Source of funding

This research received no specific grant from any funding agency in the public, commercial, or not-for-profit sectors.

## Author contribution

Conceptualization of ideas: A.A., M.A.T. Writing the initial draft: all authors. Writing—reviewing, and editing: M.A.T. Supervision: M.O.O. Funding acquisition: M.O.O. Critical review and comments: all authors. Data curation, administrative support, final approval of manuscript for publication: all authors.

## Conflicts of interest disclosure

The authors have no competing interests to declare that are relevant to the content of this article.

## Research registration unique identifying number (UIN)

1. Name of the registry is not applicable.
2. Unique identifying number or registration ID is not applicable.
3. Hyperlink to your specific registration (must be publicly accessible and will be checked) is not applicable.

## Guarantor

Malik Olatunde Oduoye.

## Data availability statement

Not applicable.

## Provenance and peer review

Not commissioned, externally peer-reviewed.
